# Emerging Perspectives From the Hearing Voices Movement: Implications for Research and Practice

**DOI:** 10.1093/schbul/sbu007

**Published:** 2014-06-13

**Authors:** Dirk Corstens, Eleanor Longden, Simon McCarthy-Jones, Rachel Waddingham, Neil Thomas

**Affiliations:** ^1^ RIAGG Maastricht, Maastricht, The Netherlands;; ^2^Institute of Psychological Sciences, University of Leeds, Leeds, UK;; ^3^ARC Centre for Excellence in Cognition and Its Disorders, Macquarie University, Sydney, Australia;; ^4^Department of Psychology, Durham University, Durham, UK;; ^5^London Hearing Voices Project, Mind in Camden, London, UK;; ^6^Brain and Psychological Sciences Research Centre, Swinburne University, Melbourne, Australia;; ^7^Monash Alfred Psychiatry Research Centre, Melbourne, Australia

**Keywords:** auditory hallucinations, service-user involvement, social psychiatry

## Abstract

The international Hearing Voices Movement (HVM) is a prominent mental health service-user/survivor movement that promotes the needs and perspectives of *experts by experience* in the phenomenon of hearing voices (auditory verbal hallucinations). The main tenet of the HVM is the notion that hearing voices is a meaningful human experience, and in this article, we discuss the historical growth and influence of the HVM before considering the implications of its values for research and practice in relation to voice-hearing. Among other recommendations, we suggest that the involvement of voice-hearers in research and a greater use of narrative and qualitative approaches are essential. Challenges for implementing user-led research are identified, and avenues for future developments are discussed.

The Hearing Voices Movement (HVM) originated in a collaboration between the Dutch social psychiatrist Marius Romme, researcher Sandra Escher, and voice-hearer Patsy Hage, in partnership with numerous individuals with lived experience of hearing voices (auditory verbal hallucinations [AVH]). This collaboration, begun in the 1980s, has since inspired an international social movement in which experts by experience (voice-hearers, family members) have worked in partnership with experts by profession (academics, clinicians, activists) to question, critique, and reframe traditional biomedical understandings of voice-hearing; develop coping and recovery frameworks; redefine the ownership of power and expertise; and promote political advocacy for the rights of those who hear voices.^1^ The development of peer support groups for voice-hearers, known as “hearing voices groups” (HVGs), are a particularly striking consequence of this movement. In England, eg, there are now over 180 groups hosted in a range of settings including child and adolescent mental health services, prisons, inpatient units, and the voluntary sector. Organized into a number of local and national networks, the success of this approach can also be seen by its diffusion in the past 20 years throughout Europe, North America, Australia, and New Zealand, emerging initiatives in Latin America, Africa, and Asia; and the success of the sixth World Hearing Voices Congress (Melbourne, Australia, 2013), which was attended by nearly 800 delegates. Within these international networks, the combined experience of voice-hearers and professionals have overseen the development of ways of working with people who hear voices that draw on the value of peer support and which help people to live peacefully and positively with their experiences.^1–3^ Given its popularity, the approaches generated by the HVM appear to offer an attractive alternative for voice-hearers who have not been fully helped by traditional approaches, who are searching for greater understanding and acceptance of their experiences, or who feel that their stories have not been heard or acknowledged.

## A Brief History of the HVM

The first article to articulate the practice and philosophy of the HVM was published over 20 years ago in this journal by Romme and Escher.^4^ Within it, they related a process of inviting 20 nonpatient voice-hearers to share insights on how they successfully coped with their experiences. These individuals were later invited to tell their stories at the first Hearing Voices Congress in 1987, and on the basis of subsequent interviews with 300 voices-hearers, Romme and Escher presented a developmental phase model of coping with voices: consecutively the (1) startling, (2) organization, and (3) stabilization phases, each of which required specific strategies and contingencies. Diverse frames of reference for voice-hearing experiences were reported: both internal (eg, psychodynamic, biomedical) and external (eg, parapsychological, mystical, and technological). Romme and Escher concluded that reducing and reifying voice-hearing to merely a pathological phenomenon was not always beneficial in respect to helping people to learn to cope with their voices. Instead, they recommended that effective practice for supporting distressed individuals should involve trying to understand the voice-hearer’s frame of reference, supporting them to change their relationship with their voices, and promoting the valuable role of peer support for decreasing social isolation and stigma.

For voice-hearers, this paradigm provided an attractive alternative or adjunct to traditional psychiatric approaches, often summarized as “trying to silence the voices”—both of the voices themselves,^5^ and of the voice-hearer’s own voice.^6^ In recognition of the importance of this survivor voice, the HVM has held annual congresses about voice-hearing, where experts by experience shared their stories of understanding, healing, and recovery on an equal basis to those experts by profession and/or experience who present alternative perspectives. The first national Hearing Voices Network was launched in the Netherlands in the early 1990s, and the United Kingdom soon followed. Organizing peer support became an important focus of Hearing Voices Networks, and these initiatives were embraced by voice-hearers themselves as offering a safe space to explore and understand their experiences. Subsequently, a number of prominent voice-hearing activists began providing training to academics and mental health professionals (eg, Coleman, Bullimore, Dillon).

As the HVM advanced as a social and psychiatric movement, a complementary literature also began to develop. Romme and Escher’s first book, *Accepting Voices*,^7^ advocated several frames of reference and a diverse range of alternative approaches to standard practice. Simultaneously, comparative research of patient and nonpatient voice-hearers^8^ revealed that self-efficacy, anxiety, and perceived voice omnipotence (rather than voice presence per se) were important variables in determining whether individuals required psychiatric care. In the book *Making Sense of Voices*,^9^ Romme and Escher subsequently outlined a detailed assessment model of voice-hearing experiences, the Maastricht Hearing Voices Interview. This tool can be used to devise a “construct,” a type of psychological formulation that attempts to determine (1) who/what the voices represent and (2) what problems the voices represent (see also Corstens et al,^9 Longden et al,10^ and Corstens and Longden^11^). More recently, the book *Living with* *Voices* ^3^ presented 50 stories of individuals who had learned to cope successfully with their voices. Within the HVM, such testimonies are considered powerful “narrative evidence” of the success of the approach. In this respect, some authors deem “the voice-hearer” as a separate and liberating identity in the context of traditional psychiatric practice. For example, Woods^12^ has described how “The figure of ‘the voice-hearer’ comes into being through a specific set of narrative practices as an ‘expert by experience’ who challenges the authority and diagnostic categories of mainstream psychiatry”^12(p263)^ (see Boxes 1 and 2).

**Box 1 Case Vignette A**

Michael, 23-years old, had heard aggressive voices for a number of years that told him to attack other people. He grew afraid that he would be unable to control them and sought help from a psychiatrist, requesting medication to suppress his voices. During the initial assessment the author (DC) explained that voices often emerge for plausible, emotional reasons, and that voices can make sense in a person’s life. This resonated with Michael, and he begun to explore what the reasons in his own life could be. He later recounted that his voices had started at a very young age after the family moved to a new part of the city, where he felt extremely unsafe. His beloved grandfather died in that period, and after being bullied by a group of peers he became increasingly insecure. He lacked support or validation at school and had withdrawn from social life. At a young age he started martial arts and it was formulated that the aggressive voice reflected his trainer at that age. In the first weeks of treatment, the power of the voices dramatically subsided as Michael began working with the emotions that they represented (particularly aggression and low self-esteem), as well as frustrations and difficulties within his family. He now is a paid peer support worker, leads a self-help group, and is very skilled at supporting other voice-hearers who struggle with aggressive voices. He never took medication, nor was he ever admitted to a psychiatric hospital. The initial diagnosis of schizophrenia played no role in the therapeutic approach, although the classification was confirmed by a SCID-I interview by an independent interviewer.

–Adapted from Knols and Corstens^13^

**Box 2 Case Vignette B**

Nelson, a 47-year old ex-army sergeant diagnosed with schizophrenia, attended a four-day “Working with Voices” training session for voice-hearers and professionals (facilitated by DC and EL) to try and make sense of his voices. Previously he had begun to taper down his medication after he encountered the ethos of the HVM, reporting that the neuroleptics dulled his emotions and had no impact on his voices. During the course it emerged that severe childhood abuse and neglect were related to the onset of his voices, which first appeared when he was aged seven. The loss of his best friend in military action 10 years previously had triggered his first, but recurrent, psychosis. Until that time he always had been able to deal with his voices, but feelings of grief and guilt about his deceased friend (combined with being the victim of a serious sexual assault) hindered successful coping and caused an aggressive and debilitating change in the voices. Trauma had never been addressed in previous psychiatric treatment, and this was the first time he had ever been asked about the context or content of his voice-hearing experiences.

In a session we talked with one of his three voices. This voice identified itself as “Judas,” a military-type figure that encouraged Nelson to be assertive. Interestingly Judas revealed that he was “the protector of Christ” and not “the traitor of Christ.” Nelson was raised in a religious household, and this idea can be traced back to gnostic scriptures. Symbolic meaning is often hidden in voices’ presentation; and Nelson acknowledged that when Judas first appeared in his childhood, it was as a protector who helped him cope emotionally with the abuse. In recent years, Judas became aggressive and challenging whenever the second voice (a seven-year old boy called “John”) became emotionally overwhelmed. John, Judas described, couldn’t cope at all with his traumatic memories. This often happened in response to the third voice, “Mother,” who embodied Nelson’s abusive parent. During the session the relationship between Judas and John was restored through mutual understanding, and Judas pledged to support John and Nelson more positively, both in response to “Mother” and to external challenges and responsibilities. Judas also collaborated with Nelson, DC, and EL on a recovery plan. When Nelson woke up the next day, the first thing that Judas said was “good morning.” Nelson reported that this was the first time in recent memory that Judas had uttered anything pleasant or companionate.

–Adapted from Corstens, Longden, and May^14^

## Key Values of the HVM

While the HVM incorporates people with a wide range of perspectives, there are some core values to which members in general subscribe. The first is the normalizing belief that hearing voices is a natural part of the human experience. Voices themselves are not viewed as abnormal or aberrant, rather conceptualized as a meaningful and interpretable response to social, emotional, and/or interpersonal circumstances. According to this perspective, the potential for voice-hearing exists in all of us. For many voice-hearers, this is more constructive and empowering than disease-based conceptualizations that emphasize pathology and may induce stigma, reduce self-esteem, and lead to an emphasis on eliminating the experience that can be unrealistic, given the limited effectiveness and hazardous side effects associated with current pharmacological treatments.^3^ The potential for voices to be experienced in certain circumstances in any individual is borne out by studies of the effects of sensory deprivation, of events such as bereavement, trauma, and ingestion of hallucinogens^15^ and of the widespread acceptance of voices as a normal phenomenon in a number of non-Western cultures.^16^ Similarly, voices are often experienced in persons in community samples without a history of psychiatric disorder. In this respect, epidemiological studies suggest that a significant minority of the population have had an experience of hearing voices at least once in their life.^17^ Voice-hearing hence appears to be an experience that extends into the general population, suggesting that prevailing views in Western society of voices as inevitable signs of psychiatric disturbance need to be reevaluated.^18^

Secondly, diverse explanations for voices are both accepted and valued, and the HVM respects that people may draw on a range of explanations to make sense of their voices. This is consistent with widely held cultural beliefs about voices^19^ and with beliefs held by people in community samples who experience voice-hearing without a need for psychiatric help.^20^

Thirdly, and consistent with the above, voice-hearers are encouraged to take ownership of their experiences and define it for themselves. Hearing voice groups often provide a safe space for this exploration, with a multiplicity of explanations held as a key principle.^21^ Because of this, terms like “AVH” and “delusions” may evoke resistance because they represent medical discourse that may often be perceived as disempowering and potentially colonizing of the individuals’ own explanatory framework. Such perceived annexation of the voice-hearing experience is a very old phenomenon; eg, Martin Luther noted the tendency for physicians to overwrite spiritual explanations of voice-hearing with protomedical models.^22^

Fourthly, it is believed that in the majority of cases voice-hearing can be understood and interpreted in the context of life events and interpersonal narratives.^11,18,23^ Specifically, it is often reported that voices are precipitated and maintained by emotional life events that overwhelm and disempower the individual, with the content, identity, and/or onset of voices frequently corresponding to broader issues in the person’s life.^3,7,24^ Tools like the Maastricht Hearing Voices Interview, and “the construct” that is derived from it, can be employed to understand—and attempt to address and resolve—the latent conflicts that may underlie the voices’ presence.^9–11^ The claim that voice utterances are psychologically meaningful in relation to the voice-hearer’s life, rather than arbitrary content induced by disease, has a long history in both psychiatry, psychology, and philosophy, being purported by writers such as Pinel, Bleuler, Jaspers, and Laing^22^ and the HVM can be seen as a contemporary instantiation of this tradition.

Fifth, a process of accepting voices is generally regarded as more helpful than attempting to suppress or eliminate them. This involves accepting the voices as a real experience, honoring the subjective reality of the voice-hearer, and recognizing that voices are something that the voice-hearer can—with support—deal with successfully.^1,2^ Actively valuing the voices (eg, as meaningful and significant emotional experiences) exceeds basic acceptance and can feel counter-intuitive when someone hears distressing or commanding voices. In this respect, Romme and Escher propose that the voices are both the “problem” and “solution”: an attack on identity, yet an attempt to preserve it by articulating and embodying emotional pain.^25^ “Decoding” the conflicts and life problems represented by voices is often possible even when people are diagnosed with complex and chronic mental illness.^11^ However, consistent with the diversity of opinion valued by the HVM, if voice-hearers choose to take antipsychotic medications to manage or eradicate voices, this too is respected. Equally, many voice-hearers find medication to be useful in reducing emotional intensity and/or promoting sleep. However, medication is only viewed as one of many available strategies, and it is very important within this paradigm that people are supported to make their own decisions about their treatment and have the necessary information to make an informed choice.

Finally, peer support is seen as a fruitful means of helping people to make sense of and cope with their voices. Mutual support groups have a long association with the HVM, with an emphasis on group ownership rather than following a predetermined structure.^2^ Online support forums are an increasingly common feature, and the role of one-to-one peer work is also sometimes used as a means of promoting change,^26,27^ embracing principles such as those of Intentional Peer Support (table 1).^28^

**Table 1.** Key Values of the Hearing Voices Movement

1.	Hearing voices can be understood as a natural part of human experience
2.	Diverse explanations are accepted for the origins of voices
3.	Voice-hearers are encouraged to take ownership of their experiences and define it for themselves
4.	Voice-hearing can be interpreted and understood in the context of life events and interpersonal narratives
5.	A process of understanding and accepting one’s voices may be more helpful for recovery than continual suppression and avoidance
6.	Peer support and collaboration is empowering and beneficial for recovery

Although sometimes perceived as contentious and marginal in professional circles, these ideas accorded with the type of psychosocial causal explanations and treatments favored by many service-users and their families,^29^ as well as psychological perspectives on voice-hearing, and the general drive toward recovery-oriented mental health practice.

Correspondingly they have grown progressively more accepted and mainstream, with many of the HVM’s basic assumptions gathering empirical support in the last 10 years. This includes, eg, the increasing evidence for a continuum model of voices and similar experiences^30^; the robust associations between voices and traumatic; adversarial life events in both clinical and nonclinical populations^31–34^; the suggestion that voice content is psychologically significant and meaningful^3,11,35^; the finding that greater levels of emotional suppression are associated with more frequent and troublesome voice-hearing experiences^36^; the commonality in structural voice characteristics between psychotic patients, nonpsychotic patients, and nonclinical groups^18,22,37^; comparable patterns of functional activation in clinical and nonclinical voice-hearers^38^; links between voice-hearing and mental health problems being primarily determined by an individual’s interpretation of and/or emotional response to their voices^3,39,40^; and the development of relational approaches to voice-hearing within cognitive behavioral therapy (CBT).^41,42^

The paradigm shift from voice-hearing as a generic symptom to an understanding of voices as a meaningful experience that can direct personal change and recovery has appealed to many voice-hearers, workers, and family members; ultimately creating a shared identity, a new language, and practice of hope. As Woods^12^ has stated: “Twenty-five years after the Hearing Voices Movement first created the space for people to discuss voices, ‘the voice-hearer’ has become established as an identity people can adopt, inhabit, and mobilize in order to lay claim to a view of voice-hearing as meaningful in the context of people’s lives. The challenge, perhaps, for the next quarter century is for the mental health professions fully to recognize this claim and its potentially radical implications.”^12(p268)^

## Challenges and Implications for Research

Although the origins of the HVM were based in collaborative research led by Romme and Escher, the HVM primarily developed as survivor-led: prioritizing advocacy for the reform of mental health service provision, promoting the rights of voice-hearers, and working collaboratively with individuals to explore the meaning of their voices and work toward recovery. Framing voice-hearers as a marginalized group, the HVM stands alongside other social movements that have prioritized personal experience and testimony as an important source of evidence,^1^ while having an uneasy relationship with the traditional research methods used within the medical and social sciences.^2^ As such, its history has diverged from the mainstream research agenda. However, recent years have seen a resurgence of empirical research both within the HVM and collaborations with academic and clinical allies.^2,11,19,43^ This has opened up a debate as to whether formal research has a place in the HVM, and, if it does, what type of research HVM members believe to be valuable.

The following are key issues relating to research highlighted in recent discussions by representatives of Intervoice (the international coordinating body for the HVM and allied Hearing Voices Networks) at the meeting of the International Consortium for Hallucinations Research (ICHR) in the United Kingdom in September 2013, and a continuing dialogue by ICHR members attending the 2013 World Hearing Voices Congress with experts by experience in this forum.

### Participation and Collaboration

Consonant with its collaborative origins, as well as contemporary developments in service user/survivor-led research, the HVM approach necessitates full participation of experts by experience at all stages. This includes setting questions and developing methodologies.^44^ Depathologization and collaboration between different disciplinary and experiential backgrounds are also cited as desirable trends.^45^ More fundamentally, “nothing about us without us” is a broad principle in respecting the rights of those who are marginalized by their experiences in decision making related to them. In research terms, this means a priority for researchers is to forge collaborative relationships with voice-hearers to facilitate their active involvement in the design and conduct of research. This requires an investment of resources into training individuals with lived experience to understand research methodology and practice in order to meaningfully contribute to both study design and interpretation. It also requires funders to recognize the value of collaborative research and to consider prioritizing initiatives that demonstrate involvement. To date, this approach has been most successfully adopted in the *Hearing the Voice* Project at Durham University in the United Kingdom, where people with lived experience are active collaborators in a multidisciplinary program of research. We believe this is a model that could be adopted in other research centers with a particular interest in voice-hearing.

### Research Terminology

As discussed previously, the language commonly used in voice-hearing research can provoke resistance in people with lived experience of hearing voices.^22^ While researchers may feel that the term “AVH” is neutral and technically descriptive terminology, voice-hearers themselves may perceive it as loaded with the assumption that voices are not real and/or that they are best explained in a biomedical manner. Equally, describing voices as “symptoms” and unusual beliefs as “delusions” can convey the belief that these phenomena are induced by illness, which may exclude those with nonmedical frames of reference for their experiences. Knowledge differences are difficult to bridge, yet acknowledgement of different expertise is necessary. While finding truly neutral and descriptive language that is not based within the illness paradigm is a challenge, working toward this may help build common ground and engage people in research who would normally avoid it.

### Researching Peer Support

Peer support is a core element of therapeutic work advocated by the HVM. HVGs arose out of the HVM as a social movement and were developed and implemented primarily by mental health service-users and community workers rather than clinician researchers. As such, they were intended to facilitate shared experience and the empowerment of group members (rather than for a therapeutic effect per se), and the anecdotal evidence of benefits has yet to generate a comprehensive and systematic appraisal of effectiveness. An randomized clinical trial (RCT) design could feasibly be used to approximate simplified types of HVGs if key elements of their principles were distilled. This might be utilized to determine whether peer support in conjunction with routine care results in greater short-term changes in the subjective impact of voices and personal recovery than routine care alone (eg, HVG vs wait-list control) and whether changes arise, which are distinct from those provided by professional support (eg, HVG vs a psychoeducation class), such as on personal recovery-relevant constructs like hope, internalized stigma, and perceived isolation.

However, it is important to acknowledge the inherent challenges in utilizing RCT methodology to evaluate these groups. First, a core part of the philosophy of HVGs is that the responsibility for group content is owned and developed by the members themselves rather than using a predetermined manualized structure. Second, in line with the HVM’s emancipatory philosophy, an open format is advocated wherein members are able to join and leave the group at any time, presenting research challenges in participant enrolment and tracking. Third, many of the benefits of HVGs are thought to arise from longer term membership, requiring a long period of intervention delivery and follow-up to capture, less well-suited to typical funding time frames available for randomized trials. Fourth, groups often move through phases in their development, with established groups benefiting from a number of more experienced members assuming leadership roles as the group matures. This could not be captured if evaluating a series of newly formed groups. Fifth, in addition to there being a lack of service-user-developed outcome measures, the individual process of recovery means that many of the changes reported by group members may not be captured by standardized measures. Sixth, the process of randomization to control vs intervention conflicts with one of the principles of self-help,^2^ that everybody must have access to self-help groups. Because of these considerations, there is debate within the HVM as to whether the compromises required to run an RCT would be so great that they would lose core features of HVGs as they are run in practice.

In addressing the need for examining the effect of groups, it may be that a range of complimentary methodologies are needed. For example, qualitative data could investigate members’ experiences of HVGs and any impact on their quality of life. It may also explore any changes (positive or negative) in the way they understand, deal with, and feel about the voices they hear. Compatible quantitative instruments could assess relevant variables like quality of life, self-esteem, depression and anxiety, internalized stigma, and social isolation—alongside more nuanced measurements of voice-hearing, suggested in the following section. Other designs might include taking elements of methods used in HVGs, such as sharing peer stories, and applying them in an individualized format; in this respect, a pilot RCT of a one-to-one peer support intervention is currently underway in Australia. Finally, multiple baseline studies and experience sampling could probably be less disruptive for group processes and pay more respect to individual differences.

### Evaluation Across the Spectrum of Therapeutic Interventions

A crucial issue in evaluating all therapeutic strategies for voices is defining the most appropriate outcomes. From the HVM perspective, voice-hearing is understood as a meaningful experience that should be validated and acknowledged. Intervention research that is aimed solely at eliminating voice presence may be perceived as substantiating at first sight, but in practice, the goal of eradication is typically not reliably achieved—neither by psychosocial interventions (Thomas et al, this issue) nor by pharmacotherapy.^46^ Being able to experience voice-hearing with reduced negative impact on subjective well-being and independent functioning; improving relationships with particular voices; and developing a sense of pride, peacefulness, and empowerment in one’s identity as a voice-hearer may be equally important intervention aims.

Such considerations have important implications for how we measure outcomes. In studies of both pharmacological and psychosocial interventions for voices, outcome has been operationalized primarily in terms of symptom levels. Most generally, trials consider whether people with a schizophrenia diagnosis are rated as demonstrating short-term reductions in overall psychotic symptomatology on measures such as the Positive and Negative Syndrome Scale.^47^ When voices are focused on more specifically, the most widely used outcome variable has been the total score on the Psychotic Symptom Rating Scales,^48^ an overall severity index on which the majority of component items correspond to structural voice characteristics (eg, frequency, loudness, duration, location, and content) rather than subjective adaptation. There has been some recent development of service-user-informed measures of the subjective impact of psychosis,^49^ CBT for psychosis outcome,^50^ and personal recovery.^51^ However, at present, there are no outcome measures of voice experiences that have been informed by consultation with voice-hearers themselves. Identifying the domains of outcome seen as important by voice-hearers, and developing a measure of these, should be a priority for intervention research. Dimensional measurement tools may be particularly helpful in this regard because they are capable of capturing nuanced changes in a person’s relationship with their voices and the impact of this on their well-being.

There are also challenging issues for outcome research associated with the time-course of the process of learning to live with voice-hearing. Clinical trial methodologies derived from pharmacotherapy research are primarily suited to examining easily measurable effects occurring over a period of days to weeks, rather than assessing longer term processes of recovery and adaptation. Additionally, it should also be recognized that personal recovery is nonlinear. In this respect, the HVM perspective is that recurrences and relapses may represent opportunities for learning and growth, sometimes being an integral part of an individual’s recovery journey and not an inevitably adverse outcome. If this is true, we may need to reconsider assumptions about positive and negative outcomes within practice trials. Indeed, the possibility that short-term clinical changes might not capture the full picture was recently brought into focus by the finding that maintenance antipsychotic medication following first-episode psychosis may be associated with benefits in terms of relapse prevention over 2 years but poorer functional outcome at 7-year follow-up.^52^ In addition to longitudinal research examining recovery over a number of years, qualitative methodologies in which personal experiences of recovery can be considered in more detail are particularly valuable for gathering a more complete picture of individual recovery. This may enable the development of more sensitive and appropriate tools for larger scale outcomes research.

Finally, in addition to being of importance in determining how outcomes are measured, this has further relevance for the types of interventions studied. As well as peer support, therapeutic interventions based on the key values and assumptions of the HVM, and which are employed by many of its proponents, include the Maastricht Hearing Voices Interview,^25^ formulating voices with a construct,^9–11^ dialoguing with voices,^14^ sharing stories, and individual peer support/recovery work.^1–3^ Studies experimentally examining the effects of such methods are also important for contributing to the evidence base of outcome research.

## Challenges and Implications for Practice

The HVM understands voice-hearing as a common experience, not inevitably pathological in itself but rather part of the diversity of the human condition.^15,17,19^ It encourages people to define their own experiences and, if they find their voices distressing, to seek personal interpretations and holistic coping strategies. From the HVM perspective, even intense and seemingly bizarre phenomena can be meaningfully interpreted and understood in the context of someone’s interpersonal narrative. When mental health professionals meet those who are extremely overwhelmed and confused by their experiences, retaining the perspective that these experiences make sense can be a challenge in itself. However, consistent with the principles of much CBT practice—in which it is customary to emphasize the continuum between voice-hearing and more familiar mental events, like intrusive thoughts^53^—situating voices as an intelligible human experience may prove reassuring, reduce shame, and stigma, and promote a positive a positive therapeutic alliance.^3^

It is important to acknowledge that our current methods of supporting voice-hearers within psychiatry are often based on limited evidence. The evidence for the long-term effectiveness of pharmacotherapy, the dominant treatment for psychosis, is not well substantiated^52,54,55^ and its more general hazards and evidential limitations for voices specifically are insufficiently acknowledged.^22,56^ Equally, many interventions developed within the HVM (eg, voice dialoguing and the construct) lack a robust evidence base or are simply difficult to research (eg, HVGs). This practice-research gap, as outlined above, requires careful exploration and investigation—with an attendant rethinking of the kinds of evidence that we use and the outcomes we are evaluating. While this gap exists, however, it is important that we use a careful synthesis of social, psychological, and biological knowledge to create care pathways for distressed voice-hearers that meet their individual needs and preferences. Given that alternative explanatory models are highly validated,^15,19,57^ it is important to consider cooperation with healers from nonpsychiatric perspectives to assist those who understand their voices in the context of their culture of spirituality. Furthermore, given the high prevalence of trauma among those who experience distressing voices,^11,31–34^ and that the role of trauma remains significantly underestimated in psychosis more generally,^58–60^ we need to pay greater attention to developing trauma-informed practice in all services.

It is well recognized that hope and optimism is a key aspect of recovery from severe mental health problems.^61,62^ As such voice-hearers may benefit from more positive information about their experiences and prognosis, as well as exposure to positive role models. Diagnostic classification and its often stigmatizing effects do not do justice to the uncertainty of prognosis nor the potential inefficiency and risks of routine pharmacological approaches.^52,63^ Prescription of neuroleptics should be cautious and postponed,^64^ especially given the availability of sound alternatives with more modest, short-term roles for medication such as Open Dialogue^65^ and Soteria.^66^ Eradicating voices pharmacologically, even if patients understandably request this, is not always a realistic treatment goal.^54^ Limitations in the existing evidence base for neuroleptic medication for voices have been highlighted,^22,56^ and it has been argued that there is not enough robust evidence to support the routine administration of such medication for voice-hearers (who are diagnosed with psychosis).^56^

Furthermore, voices may reflect information that can be used to inform recovery planning.^18^ As such, silencing them could provide short-term benefit in the sense that perceived threat is decreased but in the long-term could forfeit the opportunity to discover and explore social-emotional issues that can be utilized therapeutically as a focus for personal change.^9–11^ Individuals who are distressed by their voices may potentially benefit most from approaches that incorporate acceptance and normalization; a focus on coping with emotions as much as coping with the voices themselves; the development of a helpful and interpersonally coherent narrative; and, potentially, valuing the voices as “messengers” that may be hard to hear, but can represent opportunities for self-knowledge and psychological growth. Recovery journeys are personal, variable, and mutable, yet voice-hearers do recover and are able to integrate the voices into their lives.^3,67,68^

## Challenges for the HVM

This article shares some strong opinions on voice-hearing and the need to change contemporary thinking and practice for understanding voices. Likewise, it is necessary for the HVM to subject its own principles and practice to the same scrutiny as those it critiques and to ask itself challenging questions to avoid simply idealizing its own ideas. One of the most fundamental principles of the HVM is that voices have personal meaning, “messengers” that embody and represent real-world issues. However, it also needs to consider whether it is possible that some instances of voice-hearing have no biographical relevance and are better accounted for using biomedical models.

Given that the roots of the HVM combine social action and protest alongside attempts to create more therapeutic options for distressed voice-hearers, it is important to untangle these 2 differing goals. Activities based on social action (eg, the gathering together of marginalized people to share experiences and exchange mutual support) need little more evidence than the fact people find them helpful and empowering, and choose to attend. However, activities with intended therapeutic goals that are provided within clinical services require more robust and scientific evaluation to assess their effectiveness and facilitate their improvement. Claims that have been fundamental to the origin of the HVM, but are based on limited evidence (including the 3-stage model,^69^ and the idea of voices as originating in social-emotional conflicts), also require formal testing using rigorous designs if they are to be more than an ideology.

The HVM’s recognition of the importance of personal narrative in recovery, and the problems inherent in identifying with a lifelong diagnosis such as schizophrenia, has led it to embrace the identity of “voice-hearer” as a liberating alternative. The HVM needs to understand more about people’s diverse experiences of this label, as much as any diagnostic one. Is it, eg, confining and uncomfortable for some? As noted above, the language used within mainstream psychiatric practice can be alienating. Equally, the HVM needs to reflect on its use of language and labels and consider whether the terminology it employs is creating an alternative discourse that has the potential to define and limit experience in the same way as more medical frameworks.^12^ Within HVM conferences, there has been a move toward people describing embodied experience of voices and voices as “parts” of the voice-hearer themselves. These are multisensory experiences that the word “voice-hearing” does not adequately capture. The HVM also needs to be aware of, and responsive to, people’s experience of visions and other sensations above and beyond auditory phenomena.

In line with its radical roots, there is tension within the HVM as to whether it should focus its efforts on developing approaches within established systems (eg, utilizing the skills of qualified practitioners and established approaches, such as CBT) or create alternatives outside of this. Equally, there is also the need to consider whether it focuses on supporting the individual voice-hearer or turn its attention toward the very real issues of systemic adversity, abuse, and injustice that research implicates in the origins of distressing voices. The HVM’s dual focus on human rights, emancipation, and societal change on one hand, and support, treatment, and healing on the other, could appear confusing to some—especially when the better known aspects of its work are more aligned with the latter. It may be important to articulate these differing aspects of the HVM more clearly, especially in regard to developing an evidence base and embedding approaches within support services.

## Conclusions

The HVM promotes empowerment and validation for voice-hearers and emphasizes a fusion of individual understanding and the fellowship and solidarity of peer support as important ingredients for successful recovery. In addition to critical reflection on its own practice and philosophies, the HVM perspective also identifies challenges for clinical research and practice. This includes the provision of choice, normalization, and hopeful information, as well as an urgent need for genuine collaboration with voice-hearers in research, not only for creating an alternative research climate but also for providing opportunities in the recovery journey.

## Funding

Macquarie University Research Fellowship and Wellcome Trust Award (WT098455 to S.M.J.).

## References

[1] Longden E Corstens D Dillon J Recovery, discovery and revolution: the work of Intervoice and the hearing voices movement. In: Coles S Keenan S Diamond B, eds. Madness Contested: Power and Practice. Ross-on-Wye, UK: PCCS; 2013:161–180

[2] Dillon J Hornstein GA Hearing voices peer support groups: a powerful alternative for people in distress. Psychosis Psychol Soc Integr Appr. 2013;5:286–295

[3] Romme M Escher S Dillon J Corstens D Morris M, eds. Living with Voices: Fifty Stories of Recovery. Ross-on-Wye, UK: PCCS; 2009

[4] Romme MA Escher AD Hearing voices. Schizophr Bull. 1989;15:209–216 2749184 10.1093/schbul/15.2.209

[5] Kapur S Psychosis as a state of aberrant salience: a framework linking biology, phenomenology, and pharmacology in schizophrenia. Am J Psychiatry. 2003;160:13–23 12505794 10.1176/appi.ajp.160.1.13

[6] McCabe R Heath C Burns T Priebe S Engagement of patients with psychosis in the consultation: conversation analytic study. BMJ. 2002;325:1148–1151 12433765 10.1136/bmj.325.7373.1148 PMC133454

[7] Romme M Escher S Accepting Voices. London, UK: Mind; 1993

[8] Honig A Romme MA Ensink BJ Escher SD Pennings MH deVries MW Auditory hallucinations: a comparison between patients and nonpatients. J Nerv Ment Dis. 1998;186:646–651 9788642 10.1097/00005053-199810000-00009

[9] Corstens D Escher S Romme M Accepting and working with voices: the Maastricht approach. In: Moskowitz A Schäfer I Dorahy MJ, eds. Psychosis, Trauma and Dissociation: Emerging Perspectives on Severe Psychopathology. Oxford, UK: Wiley-Blackwell; 2008:319–331

[10] Longden E Corstens D Escher S Romme M Voice hearing in biographical context: a model for formulating the relationship between voices and life history. Psychosis Psychol Soc Integr Appr. 2012;4:224–234

[11] Corstens D Longden E The origins of voices: links between life history and voice hearing in a survey of 100 cases. Psychosis Psychol Soc Integr Appr. 2013;5:270–285

[12] Woods A The voice-hearer. J Ment Health. 2013;22:263–270 23691942 10.3109/09638237.2013.799267 PMC3836250

[13] Knols M Corstens D Tuning in: a story by a patient and a therapist about making sense of voices. Ment Health Today. 2011; Nov–Dec:28–32 22216606

[14] Corstens D Longden E May R Talking with voices: exploring what is expressed by the voices people hear. Psychosis Psychol Soc Integr Appr. 2012;4:95–104

[15] Watkins J Voice Hearing: A Common Human Experience. Melbourne, Australia: Michelle Anderson; 2008

[16] al-Issa I The illusion of reality or the reality of illusion. Hallucinations and culture. Br J Psychiatry. 1995;166:368–373 7788129 10.1192/bjp.166.3.368

[17] Beavan V Read J Cartwright C The prevalence of voice-hearers in the general population: a literature review. J Ment Health. 2011;20:281–292 21574793 10.3109/09638237.2011.562262

[18] Johnstone L Voice hearers are people with problems, not patients with illnesses. In: Romme M Escher S, eds. Psychosis as a Personal Crisis: An Experience-Based Approach. London, UK: Routledge; 2011:27–36

[19] McCarthy-Jones S Waegeli A Watkins J Spirituality and hearing voices: considering the relation. Psychosis. 2013;5:247–258 24273597 10.1080/17522439.2013.831945 PMC3827668

[20] Daalman K Boks MP Diederen KM The same or different? A phenomenological comparison of auditory verbal hallucinations in healthy and psychotic individuals. J Clin Psychiatry. 2011;72:320–325 21450152 10.4088/JCP.09m05797yel

[21] Dillon J Longden E Hearing voices groups: creating safe spaces to share taboo experiences. In: Romme M Escher S, eds. Psychosis as a Personal Crisis: An Experience Based Approach. London, UK: Routledge; 2011:129–139

[22] McCarthy-Jones S Hearing Voices: The Histories, Causes and Meanings of Auditory Verbal Hallucinations. Cambridge, UK: Cambridge University Press; 2012

[23] Romme M Morris M The recovery process with hearing voices: accepting as well as exploring their emotional background through a supported process. Psychosis Psychol Soc Integr Appr. 2013;5:259–269

[24] Longden E Madill A Waterman MG Dissociation, trauma, and the role of lived experience: toward a new conceptualization of voice hearing. Psychol Bull. 2012;138:28–76 22082488 10.1037/a0025995

[25] Romme M Escher S Making Sense of Voices. London, UK: Mind; 2000

[26] Longden E Dillon J The hearing voices movement. In: Cromby J Harper D Reavey P, eds. Psychology, Mental Health and Distress. Basingstoke, UK: Palgrave-Macmillan; 2013:151–156

[27] May R Longden E Self-help approaches to hearing voices. In: Larøi F Aleman A, eds. Hallucinations: A Guide to Treatment and Management. Oxford, UK: Oxford University Press; 2010.

[28] Mead S Hilton D Curtis L Peer support: a theoretical perspective. Psychiatric Rehab J. 2001;5:134–141 10.1037/h0095032 11769979

[29] Read J Magliano L Beavan V Public beliefs about the causes of “schizophrenia”: bad things happen and can drive you crazy. In: Read J Dillon J, eds. Models of Madness: Psychological, Social and Biological Approaches to Schizophrenia. London, UK: Routledge; 2013:143–156

[30] Johns LC Nazroo JY Bebbington P Kuipers E Occurrence of hallucinatory experiences in a community sample and ethnic variations. Br J Psychiatry. 2002;180:174–178 11823331 10.1192/bjp.180.2.174

[31] Bentall RP Wickham S Shevlin M Varese F Do specific early-life adversities lead to specific symptoms of psychosis? A study from the 2007 the Adult Psychiatric Morbidity Survey. Schizophr Bull. 2012;38:734–740 22496540 10.1093/schbul/sbs049 PMC3406525

[32] Read J van Os J Morrison AP Ross CA Childhood trauma, psychosis and schizophrenia: a literature review with theoretical and clinical implications. Acta Psychiatr Scand. 2005;112:330–350 16223421 10.1111/j.1600-0447.2005.00634.x

[33] McCarthy-Jones S Voices from the storm: a critical review of quantitative studies of auditory verbal hallucinations and childhood sexual abuse. Clin Psychol Rev. 2011;31:983–992 21736865 10.1016/j.cpr.2011.05.004

[34] Varese F Smeets F Drukker M Childhood adversities increase the risk of psychosis: a meta-analysis of patient-control, prospective- and cross-sectional cohort studies. Schizophr Bull. 2012;38:661–671 22461484 10.1093/schbul/sbs050 PMC3406538

[35] Beavan V Read J Hearing voices and listening to what they say: the importance of voice content in understanding and working with distressing voices. J Nerv Ment Dis. 2010;198:201–205 20215997 10.1097/NMD.0b013e3181d14612

[36] Badcock JC Paulik G Maybery MT The role of emotion regulation in auditory hallucinations. Psychiatry Res. 2011;185:303–308 20678808 10.1016/j.psychres.2010.07.011

[37] Slotema CW Daalman K Blom JD Diederen KM Hoek HW Sommer IE Auditory verbal hallucinations in patients with borderline personality disorder are similar to those in schizophrenia. Psychol Med. 2012;42:1873–1878 22336487 10.1017/S0033291712000165

[38] Diederen KM Daalman K de Weijer AD Auditory hallucinations elicit similar brain activation in psychotic and nonpsychotic individuals. Schizophr Bull. 2012;38:1074–1082 21527413 10.1093/schbul/sbr033 PMC3446229

[39] Mawson A Cohen K Berry K Reviewing evidence for the cognitive model of auditory hallucinations: The relationship between cognitive voice appraisals and distress during psychosis. Clin Psychol Rev. 2010;30:248–258 20071062 10.1016/j.cpr.2009.11.006

[40] Andrew EM Gray NS Snowden RJ The relationship between trauma and beliefs about hearing voices: a study of psychiatric and non-psychiatric voice hearers. Psychol Med. 2008;38:1409–1417 18177529 10.1017/S003329170700253X

[41] Chin JT Hayward M Drinnan A “Relating” to voices: Exploring the relevance of this concept to people who hear voices. Psychol Psychother. 2009;82:1–17 18573230 10.1348/147608308X320116

[42] Hayward M Overton J Dorey T Denney J Relating therapy for people who hear voices: a case series. Clin Psychol Psychother. 2009;16:216–227 19455717 10.1002/cpp.615

[43] Beavan V Towards a definition of “hearing voices”: a phenomenological approach. Psychosis Psychol Soc Integr Appr. 2011;3:63–73

[44] Neil ST Price J Pitt L Working together: service users and researchers in psychosis research. Psychosis Psychol Soc Integr Appr. 2013;5:306–316

[45] Schrader S Illuminating the heterogeneity of voices in a multiple perspectives research paradigm. Psychosis Psychol Soc Integr Appr. 2013;5:216–225

[46] Lepping P Sambhi RS Whittington R Lane S Poole R Clinical relevance of findings in trials of antipsychotics: systematic review. Br J Psychiatry. 2011;198:341–345 21525517 10.1192/bjp.bp.109.075366

[47] Kay SR Opler LA Lindenmayr JP The Positive and Negative Syndrome Scale (PANSS): rationale and standardisation. Br J Psychaitry.1989;(suppl 7):59–67 2619982

[48] Haddock G McCarron J Tarrier N Faragher EB Scales to measure dimensions of hallucinations and delusions: the psychotic symptom rating scales (PSYRATS). Psychol Med. 1999;29:879–889 10473315 10.1017/s0033291799008661

[49] Haddock G Wood L Watts R Dunn G Morrison AP Price J The Subjective Experiences of Psychosis Scale (SEPS): psychometric evaluation of a scale to assess outcome in psychosis. Schizophr Res. 2011;133:244–249 22000941 10.1016/j.schres.2011.09.023

[50] Greenwood KE Sweeney A Williams S CHoice of Outcome in Cbt for psychosEs (CHOICE): the development of a new service user-led outcome measure of CBT for psychosis. Schizophr Bull. 2010;36:126–135 19880823 10.1093/schbul/sbp117 PMC2800145

[51] Neil ST Kilbride M Pitt L The questionnaire about the process of recovery (QPR): a measurement tool developed in collaboration with service users. Psychosis Psychol Soc Integr Appr. 2009;1:145–155

[52] Wunderink L Nieboer RM Wiersma D Sytema S Nienhuis FJ Recovery in remitted first-episode psychosis at 7 years of follow-up of an early dose reduction/discontinuation or maintenance treatment strategy: long-term follow-up of a 2-year randomized clinical trial. JAMA Psychiatry. 2013;70:913–920 23824214 10.1001/jamapsychiatry.2013.19

[53] Garrett M “Normalizing” the voice hearing experience: the continuum between auditory hallucinations and ordinary mental life. In: Larøi F Aleman A, eds. Hallucinations: A Guide to Treatment and Management. Oxford, UK: Oxford University Press; 2010: 183–204

[54] Sanjuan J Aguilar EJ Artigas F Pharmacological treatment of hallucinations. In: Laroi F Aleman A, eds. Hallucinations: A Guide to Treatment and Management. Oxford, UK: Oxford University Press; 2010:9–28

[55] Harrow M Jobe TH Faull RN Do all schizophrenia patients need antipsychotic treatment continuously throughout their lifetime? A 20-year longitudinal study. Psychol Med. 2012;42:2145–2155 22340278 10.1017/S0033291712000220

[56] Corstens D Longden E Rydinger B Bentall R van Os J Treatment of hallucinations: a comment. Psychosis Psychol Soc Integr Appr. 2013;5:98–102

[57] Caroll M Am I Going Mad? The Unsettling Phenomena of Spiritual Evolution. Kinglake, Australia: Inner Peace Publishing; 2009

[58] Read J Bentall RP Negative childhood experiences and mental health: theoretical, clinical and primary prevention implications. Br J Psychiatry. 2012;200:89–91 22297585 10.1192/bjp.bp.111.096727

[59] Read JP Wardell JD Colder CR Reciprocal associations between PTSD symptoms and alcohol involvement in college: a three-year trait-state-error analysis. J Abnorm Psychol. 2013;122:984–997 24364601 10.1037/a0034918 PMC4040515

[60] Thomas P Longden E Madness, childhood adversity and narrative psychiatry: caring and the moral imagination. Med Humanit. 2013;39:119–125 23748151 10.1136/medhum-2012-010268

[61] Resnick SG Fontana A Lehman AF Rosenheck RA An empirical conceptualization of the recovery orientation. Schizophr Res. 2005;75:119–128 15820330 10.1016/j.schres.2004.05.009

[62] McCarthy-Jones S Marriott M Knowles RE Rowse G Thompson AR What is psychosis? A meta-synthesis of inductive qualitative studies exploring the experience of psychosis. Psychosis. 2013;5:1–16

[63] Weinmann S Aderhold V Antipsychotic medication, mortality and neurodegeneration: the need for more selective use and lower doses. Psychosis Psychol Soc Integr Appr. 2010;2:50–69

[64] Bola JR Lehtinen K Cullberg J Ciompi L Psychosocial treatment, antipsychotic postponement, and low-dose medication strategies in first-episode psychosis: a review of the literature. Psychosis Psychol Soc Integr Appr. 2009;1:4–18

[65] Seikkula J Alakare B Aaltonen J Five years experiences of first-episode non-affective psychosis in Open Dialogue approach: treatment principles, follow-up outcomes and two case analyses. Psychotherapy Res. 2006;16:214–228

[66] Calton T Ferriter M Huband N Spandler H A systematic review of the Soteria paradigm for the treatment of people diagnosed with schizophrenia. Schizophr Bull. 2008;34:181–192 17573357 10.1093/schbul/sbm047 PMC2632384

[67] Dillon J The tale of an ordinary little girl. Psychosis Psychol Soc Integr Appr. 2010;2:79–83

[68] Longden E Learning From the Voices in My Head. New York, NY: TED Books; 2013

[69] Milligan D McCarthy-Jones S Winthrope A Dudley R Time changes everything? A qualitative investigation of the experience of auditory verbal hallucinations over time. Psychosis. 2013;5:107–118

